# More than a feeling: emotional regulation strategies for simulation-based education

**DOI:** 10.1186/s41077-024-00325-z

**Published:** 2024-12-31

**Authors:** Vicki R. LeBlanc, Victoria Brazil, Glenn D. Posner

**Affiliations:** 1 Department of Innovation in Medical Education, Faculty of Medicine, University of Ottawa, 850 Peter Morand Crescent, Room 102A, Ottawa, ON K1G 5Z3 Canada; 2 Faculty of Health Sciences and Medicine, Bond University, Gold Coast, QLD Australia; 3 University of Ottawa Skills & Simulation Centre, The Ottawa Hospital, Civic Campus, Loeb Research Building, 1st floor, 725 Parkdale Ave., Ottawa, ON K1Y 4E9 Canada

**Keywords:** Emotion, Interpersonal emotional regulation

## Abstract

Simulation-based education often involves learners or teams attempting to manage situations at the limits of their abilities. As a result, it can elicit emotional reactions in participants. These emotions are not good or bad, they simply are. Their value at any given moment is determined by their utility in meeting the goals of a particular situation. When emotions are particularly intense, or a given emotion is not aligned with the situation, they can impede learners’ ability to engage in a simulation activity or debriefing session, as well as their ability to retain knowledge and skills learned during the session. Building on existing guidance for simulation educators seeking to optimize the learning state/readiness in learners, this paper explores the theory and research that underpins the practical application of how to recognize and support learners’ emotions during simulation sessions. Specifically, we describe the impact of various emotions on the cognitive processes involved in learning and performance, to inform practical guidance for simulation practitioners: (1) how to recognize and identify emotions experienced by others, (2) how to determine whether those emotional reactions are problematic or helpful for a given situation, and (3) how to mitigate unhelpful emotional reactions and leverage those that are beneficial in achieving the goals of a simulation session.

## Background

Simulation is a powerful educational tool. It allows educators to recreate many elements of the clinical world so that participants can practice aspects of patient care, followed by specific and individualized feedback. Because simulation-based education often involves learners or teams attempting to manage situations at the limits of their abilities, it can elicit emotional reactions. This emotional component can be helpful, providing learners with an occasion to recognize the impact of emotions on learning and performance, as well as practice adaptive emotional regulation strategies. In some cases, however, emotional reactions can interfere with the learning objectives. When these emotions are particularly intense, or a given emotion is not aligned with the situation, they can impede learners’ ability to engage in a simulation activity or debriefing session, as well as their ability to retain knowledge and skills learned during the session [1]. However, there is limited guidance on how to recognize and support learners’ emotions during simulation sessions.

In this paper, we describe the impact of various emotions on the cognitive processes involved in learning and performance, to inform practical guidance for simulation practitioners: (1) how to recognize and identify emotions experienced by others, (2) how to determine whether those emotional reactions are problematic or helpful for a given situation, and (3) how to mitigate unhelpful emotional reactions and leverage those that are beneficial in achieving the goals of a simulation session. Our work builds on existing guidance [2–6] and more deeply explores the theory and research that underpins the practical application.

## Emotions during simulations

**Fig. 1:**
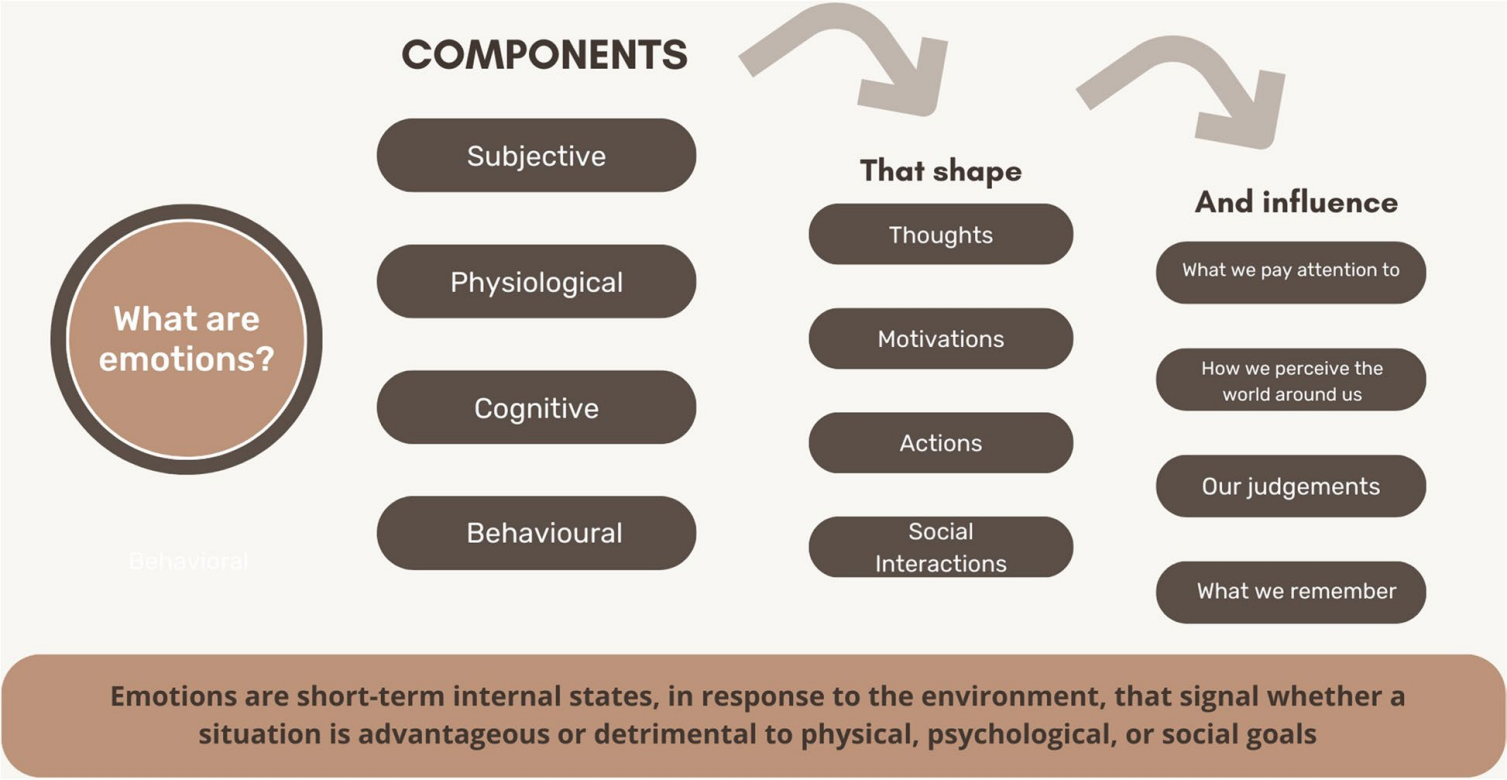
Components and impact of emotions

Emotions are short-term internal states that are accompanied by subjective experiences (e.g., feeling joy, anger, shame), physiological changes (e.g., changes in heart rate, muscular activity, skin conductivity), behavioural reactions (e.g., facial or verbal expressions, body language, and actions), as well as cognitive effects (e.g., attention, decision making, memory) [7]. Emotions are responses to the world around us: They are elicited *by* something, in reaction *to* something. Emotions differ from “moods” which are more diffuse and longer-term affective experiences that are less directly connected to a concrete stimulus (e.g., I’m feeling happy today) [7]. Emotions arise from situations that are appraised as being relevant to our needs or goals. These situations are assessed in terms of factors such as novelty, intrinsic pleasantness, predictability, whether they are beneficial or not to our needs/goals, our coping potential (ability to cope with the demands and consequences of the situation), and normative significance (compatibility with personal or social norms) [8]). In turn, the distinct emotional reactions (e.g., joy, anger, anxiety) that result from these appraisal processes have unique effects on the thought processes required for clinical performance and learning (see Fig. 1).

Many aspects of simulation sessions can trigger emotional reactions. For example, in an obstetric scenario, learning objectives may include recognizing breech presentation, counseling the patient urgently about her options, and technical skills for breech delivery. Emotions may be stimulated in this case from interacting with an anxious simulated patient, from the inherent technical challenges of the delivery, or from the perceived judgment of observers (e.g., program director observing the simulation). Depending on the emotions evoked from each of these elements, performance (and learning) may be enhanced or impaired.

A consistent feature of emotional stimuli is their ability to automatically capture our attention. Because of this, strong emotions lead to *decreased cognitive/ attentional flexibility*; that is, disengaging from one task (or problem-solving strategy) to engage in another [7]. This results in slower reaction times for information not linked to the source of the emotion. These effects are strongest for negative emotions and high-intensity ones [9]. When attention is captured by emotional stimuli that are peripheral to the learning objectives of a simulation, this can prevent the allocation of attention to the debriefing discussion and learning points. However, when emotions direct attention to one or more relevant aspects of a situation, this can improve attention to the relevant information of that situation.

Strong emotions can also *impair divided attention* [10, 11]. As such, they affect our ability to think about or do multiple things at the same time, such as keeping several pieces of information in working memory or processing information from multiple sources [12]. Strong emotions can also negatively affect the ability to *inhibit a response or action*, thus making it harder to stop an action once it has begun [7].

In the obstetrical example presented above, some residents may find that the anxiety triggered by the technical challenges helps them perform the delivery but impairs their ability to also direct attention towards effectively counseling the patient. Other residents may be unable to fully attend to what their colleagues are saying because they are preoccupied with the program director’s impressions of their performance.

Beyond directing attention, emotions also affect what is remembered. Higher arousal emotions (e.g., anger, anxiety, joy), as well as some negatively valenced emotions, are more likely to leave people *susceptible to false information* than lower arousal emotions (e.g., sadness, satisfaction) [13, 14]. In our obstetrical example, residents experiencing anger or anxiety will be less likely to verify incorrect information shared by the patient or a colleague that impacts suitability for a breech delivery, such as estimated birth weight, specific form of breech presentation, or head flexion.

Despite these complexities of emotions, they are often addressed superficially during simulation debriefings or in discussions about scenario design and delivery—as “good” or “bad”, or “emotional” or not. This superficial approach towards emotions compromises our ability to harness or mitigate emotions in pursuit of learning goals in simulation, as evidenced by variable effects of affective interventions (e.g., “reactions” phases, relaxation interventions) on learning [15, 16]. Because emotions can play an important role in learning and performance, the rest of the paper will present a deeper description of the impact of emotions and strategies to support learning in emotionally laden simulation sessions.

## Impact of emotions on thoughts and actions

Emotions serve as critical signals as to whether a situation is safe or dangerous for our physical, psychological, or social goals, as well as pleasant or disagreeable [17]. To help us achieve our goals, emotions further shape our motivations, actions, thoughts, and social interactions [7]. Because emotions arise from individual appraisal processes, the same situation can lead to different emotional reactions across individuals [8]. However, once experienced, particular emotions have predictable effects on what we pay attention to, how we interpret the world around us, the judgments we make, as well as what we remember from particular situations [18, 19]. Most importantly, emotions are neither good nor bad. Emotions simply *are*. Their value at any given moment is determined by their utility in meeting the goals of a particular situation. To determine whether emotions are beneficial for a given situation, it is important to understand how particular emotions influence thought processes. In the following section, we briefly describe the unique effects of select emotions likely to arise during simulation sessions. For more exhaustive discussions of the effects of emotions on cognitive processes, readers are encouraged to consult more in-depth reviews on the topic [7, 20, 21].

*Fear* and *anxiety* are caused by events perceived as threatening to our physical, psychological, or social goals, and for which we perceive that we don’t have the resources to manage the demands of the situation [7]. Whereas fear is typically associated with a present and specific threat, anxiety is associated with a more distant and uncertain one [22]. Because the cost of failing to detect a threat in the environment can have important consequences, the human brain has evolved to monitor for and react to possible threats in the environment. As such, potentially threatening things in our environment are prioritized by our brains [22].

These emotions disrupt selective attention, because they capture our cognitive resources, leaving less available to process a task at hand. Fear and anxiety can result in a narrowing of attention and memory, such that information central to the emotional trigger (in time, space, concept) is well remembered at the cost of peripheral information [23, 24]. These emotional states can also negatively impact working memory and can lead to more false memories from the fear/anxiety-provoking situation [25, 26]. Fear and anxiety have also been associated with alterations in reasoning, such as a higher likelihood of premature closure, greater risk perception, and decreased risk tolerance [27–31].

*Anger* results from situations where we perceive obstacles to achieving an important goal or where an undesirable event has happened, and the cause is considered controllable and external to us [7, 32]. Because it is associated with a sense of control, anger stimulates optimistic appraisals of the environment and a greater tolerance of risk. It is also associated with mental rumination; the inability to “let go” of thoughts about the misdeeds of others [33–35]. Anger has also been associated with decreases in performance on tasks that require recalling previously learned information [32].

Anger influences attention and memory by enhancing goal-relevant information processing and increases our reliance on simple cognitive processes (heuristics) [17, 36]. This results in increased use of stereotypes decreased attention to the quality of arguments, and more attention to superficial cues of messages [37]. Anger does not diminish the ability to remember events that occurred, but it decreases the ability to dismiss subsequent incorrect information about those events. Therefore, we are more likely to have false memories of an event [38]. In other words, anger leads to simpler processing approaches in order to make rapid decisions. As a result, anger can lead to increased confidence but decreased accuracy.

*Sadness* results from a perception of loss—or absence—of a reward, in circumstances interpreted as impossible/difficult to control and where the cause is unclear [7, 39, 40]. Sadness leads to a deliberative, analytical reasoning style, in the service of preventing similar losses in the future [40, 41]. As a result, it leads to a broadening of attention, less biased judgments, more accurate memories (e.g., remembering information peripheral to the sadness-provoking trigger, and resistance to false information) [25, 42], greater motivation to solve problems [42–44]*,* and more detail-oriented analyses of social information (thus, decreased susceptibility to stereotypes) [45]. When making potentially risky decisions, while sadness leads to more pessimistic thinking [40], sad individuals show a preference towards high-risk/high-reward options (selecting options that have a lower likelihood of occurring, but would give a greater reward if they do occur) [39].

*Shame* is a self-conscious emotion that is linked to the self in relation to others [46]. Shame is often accompanied by a negative self-conception and motivates a desire to escape a shame-inducing situation [47]. When feeling shame, we are prone to rumination and are more self-focused. We direct less attention towards what is external to us. As a result, we may be less able to empathize with others, have decreased working memory capacity [47], and have a decreased ability to remember information from situations in which we experience shame [48]. This contrasts with embarrassment, which is a reaction to what might be considered a “one-off” mistake or misstep that is not representative of one’s usual performance and therefore less linked to one’s self-worth [49].

Feelings of shame prompt us to disengage from our shame-inducing circumstances, either by withdrawing, attacking the other, attacking ourselves, or attempting to avoid the situation [50]. The factors determining which defensive strategy is selected by a shame-laden person are not well understood. Shame-laden individuals are particularly resistant to messages that lead to greater shame but are open to messages that lead to another emotion [51].

Unlike negatively valenced emotions each has distinct effects on thought processes, *positively valenced emotions (joy, happiness)* tend to have similar effects. Positive emotions result from the attainment of an important goal (“I successfully managed that challenging scenario”) [7], and they signal a safe environment [41]. As a result, happy people are better at detecting information that is peripheral to their focus of attention. Also, positive emotions can increase cognitive flexibility, which helps when seeking to generate solutions to a problem [52].

However, the broadened attention to positive emotions comes with a trade-off. It can lead to increased distractibility, resulting in less time spent working on tasks [42], as well as more diffuse and superficial processing of information [53]. This superficial processing can result in increased reliance on heuristics and stereotypes [7, 37, 45], an increased tendency to incorporate false information into memories, and challenges in incorporating new information with prior knowledge [45, 54].

## Recognizing emotions

If simulation educators can accurately recognize emotional states in their learners—and also sometimes in themselves—they are more likely to be able to embrace them as part of an effective learning conversation. Current strategies for identifying emotional states include learner self-reports, observed physical and behavioural manifestations, and biometric physiological markers. Not all of these strategies will help simulation educators “in the moment”, but may inform longer-term, programmatic approaches to simulation design and delivery.

A common way to identify learners’ emotions is through self-reports since emotions are part of the conscious experience [55]. Self-reports can range from simply asking individuals how they are feeling to using formalized questionnaires. This underscores the importance of the reactions phase of common debriefing frameworks [2]. The reactions phase gives participants an opportunity to name the emotions they are feeling. This creates an opportunity not only to acknowledge the emotions but to unpack them and, through the debriefing, recognize how they may have impacted performance. Importantly, learners may not feel comfortable verbally expressing their emotional state or may not have insight into their emotional state.

Simulation educators may look for other manifestations, such as facial expressions, body language or behavioural manifestations, speech, and language cues [56, 57]. Various emotions have particular sets of muscle movements that lead to distinct identifiable facial signals. These facial signals tend to be consistent across cultures, and thus be recognized cross-culturally [56]. Although display rules (who can show which emotions, to whom, and when) and symbolic gestures (head nod yes, head shake no) are socially learned and differ across cultures [56], the distinct facial signs of core emotions are fairly consistent across cultures. The display rules may influence the management of emotions, such as diminishing, exaggerating, or masking our emotions in social contexts. However, even when individuals are trying to diminish or mask their emotions, micro-expressions (very rapid facial movements) generally reveal the emotion a person is experiencing [58]. Emotional cues may also be perceived from vocal and speech patterns as well as body language, although these tend to be less specific to distinct emotions. With practice, most individuals can develop the ability to detect emotions in most circumstances. Readers seeking to further develop skills in this area are encouraged to consult more in-depth work on the topic [56, 57, 59, 60].

Physiological markers of emotions have long been of interest to researchers, and now technological advances have made the detection of physiological responses widely accessible (e.g., heart rate chest straps, smartwatches). Unfortunately, these methods are unlikely to provide meaningful information to simulation educators. Most distinct emotions do not have a specific profile of physiological patterns [61]. The one exception is stress, for which decreased heart rate variability and elevated levels of cortisol in the blood or saliva are sensitive and specific markers of stress [62, 63]. Other physiological markers (e.g., heart rate, respiration rate, skin conductance) can indicate increased physiological arousal but are not specific to valence (positive vs negative) nor distinct emotions [64–66]. These might have applications for the subset of simulation activities that are specifically focused on helping learners recognize and regulate their stress responses.

## Responding to emotions *prior to* the emotional experience

There is strong encouragement and extensive guidance for simulation educators seeking to optimize the emotional state of learners (and educators) embarking on a simulation experience [5, 67, 68]. As discussed in a previous paper, simulation educators are encouraged to be thoughtful in the inclusion of instructional design features that could trigger emotional reactions in learners [1]. In addition, supporting psychological safety encourages learners to take interpersonal risks and extend themselves, without fear of humiliation, but with an expectation of frankly discussing performance. This is a challenging task for the simulation educator. Practical behaviours—establishing rapport, active listening, providing clear expectations, tenaciously holding the “basic assumption” and employing thoughtful questioning—are widely encouraged [2–6, 69]. However, these need to be informed by attuning to learners’ expectations, experiences, and the psychosocial milieu in which their work and learning are usually conducted. Using simplistic “recipes” for establishing and maintaining psychological safety, without genuine concern (or empathy) for the learners’ emotional experiences, risks limiting psychological safety and the genuine sharing of emotional reactions by learners. Unilateral pronouncements such as “this is a safe space” from simulation educators may be problematic [70]. There is a greater risk of misaligned emotional reactions if socio-economic and social power dynamics that can affect emotional experiences (and rules around their manifestation) are ignored.

Learners should be oriented to the reality that their simulation experience will have an affective element, and that is normal and reflective of real-world practice. Every day, healthcare professionals feel emotions like anxiety about missing a diagnosis or a procedure, anger if a scenario did not play out as expected, sadness because a scenario triggers memories of a negative patient outcome, and happiness in a well-managed situation. During this orienting discussion (prebriefing), learners should be aware that emotions are potential topics of conversation in the debriefing, as a normal part of a broader discussion of performance.

## Responding to emotions *after* the emotional experience

**Fig. 2:**
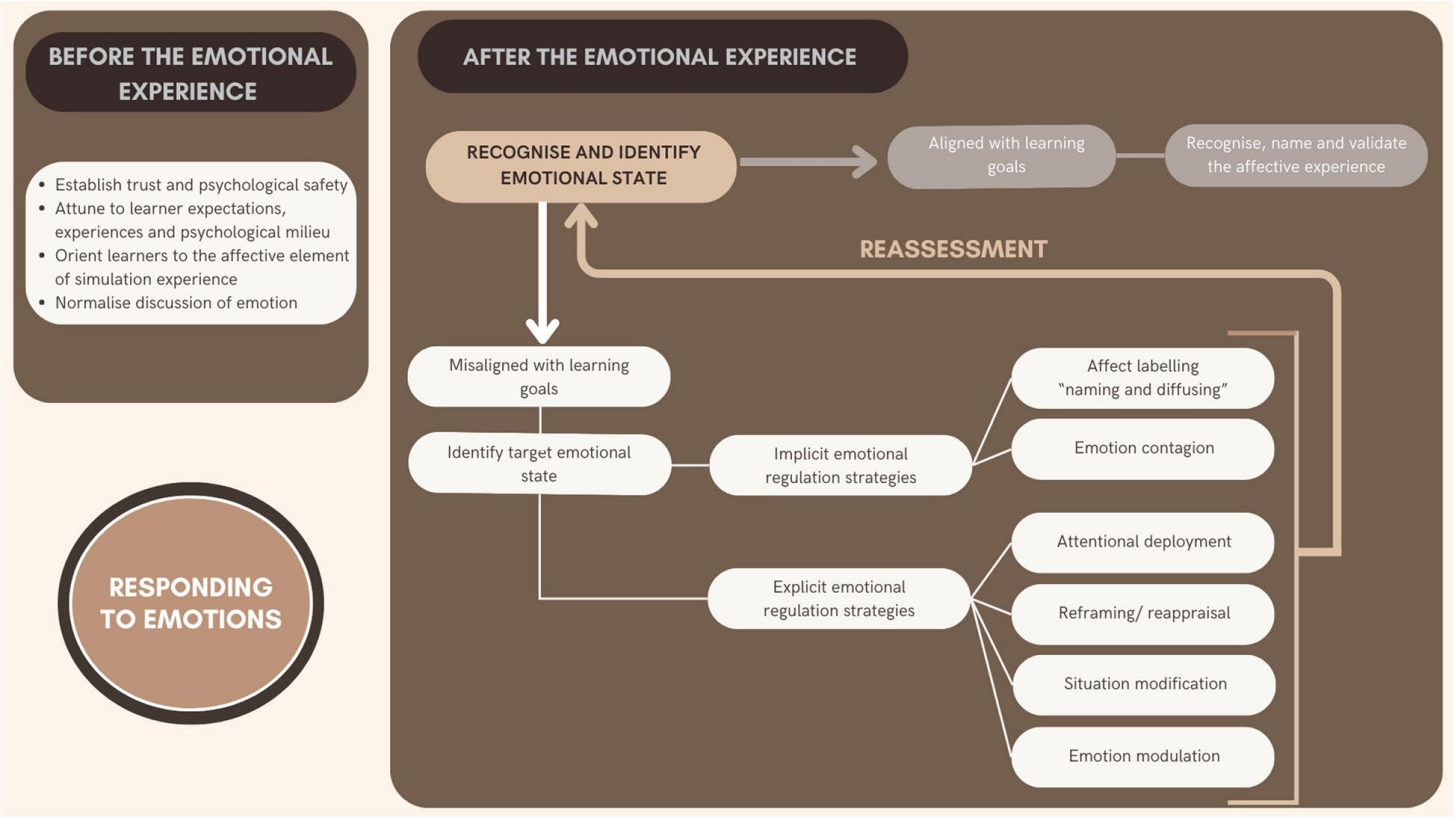
Framework for responding to emotions during simulation-based education

There is extensive guidance for simulation educators seeking to optimize the emotional state of learners to engage with and learn from, simulation debriefing [2, 4–6]. Attention to debriefing structure, thoughtful conversational techniques, and questions with a curious stance are encouraged in this guidance, as are backup strategies for “difficult debriefs”—a descriptor that encompasses many examples of maladaptive emotional responses [3]. We embrace this advice and add nuanced guidance specific to responding to and managing emotional states, after evaluating whether that emotion is conducive or not to the goals of the situation. Our general approach is reflected in Fig. 2. Although presented as a flowchart in that diagram, we emphasize the dynamic and non-linear nature of these emotional reactions and the conversations. As such, we encourage readers to embrace the core principles and strategies, rather than a rigid process.

### Identify emotional state and determine alignment with learning goals

Once simulation facilitators recognize an emotional reaction in a learner(s), they need to determine when an emotion is not aligned or is too strong to allow learning. A potential failure is the inaccurate perception of the person’s emotion, as well as regulating away from emotions that are not impediments—and are in fact conducive—to the goals of the session.

### Attempt to regulate the affective state

If the learner(s) emotional reactions are not conducive to the learning goals of a situation, there are several implicit and explicit emotional regulation strategies facilitators can use to influence a learner’s emotional state. These strategies are described in the following section. Depending on the situation, the goal can be the complete deactivation of an emotion, its reduced activation, its amplification, or even the activation of a different emotion altogether (e.g., increasing someone’s anxiety because they do not seem to recognize the potential consequences of an action) [71]. As such, emotional regulation is best defined as maintaining desirable emotional states for a particular circumstance and terminating undesirable emotional states. Rather than emotional manipulation, we are advocating for debriefers to use their skills in facilitation to create an environment where the experience that has resulted in a particular emotional reaction can be reframed or reoriented such that the emotional reaction is one more conducive to learning.

While most individuals can engage in *intra*personal emotional regulation, *inter*personal emotional regulation can be beneficial when individuals are unable to self-regulate their emotions, if they lack insight into their own emotional reaction, or if their emotional state triggers affect-congruent thinking that serves to sustain or reinforce their emotional state [72]*.* Interpersonal emotional regulation involves a *regulator* (who is engaging in the act), a *target emotional state in someone else*, and the *implementation of specific strategies or actions* to change the nature, duration, or intensity of another persons’ emotional state [73]*.*

Emotional regulation is often intentional, requires resources, and is engaged in with conscious awareness [72]. For many, the prospect of engaging in deliberate interpersonal emotional regulation may seem manipulative, or daunting and require advanced skills. However, we regularly engage in interpersonal emotional regulation in our daily lives. Individuals regulate others’ emotions more often than they regulate their own, and they put more effort into doing so [74]. Furthermore, while interpersonal emotional regulation can be cognitively and emotionally taxing, it can serve as an important social support mechanism that strengthens interpersonal bonds and increases emotional well-being in both the recipient and the provider [75, 76].

Educators seeking to successfully engage in interpersonal regulation strategies are encouraged to consider the following elements. The first is that successful interpersonal emotion regulation relies on the ability to accurately identify one’s own emotions as well as the emotions of others. Those seeking to further develop skills in this area are encouraged to consult more in-depth work on the topic [56, 57, 59, 60, 77]. Second, educators are encouraged to adopt emotional regulation strategies that are adaptive to the situation. Guidance on the application of various strategies in different contexts is presented in the next section. Third, educators are encouraged to practice (e.g., role-playing with peers and receiving feedback) the various emotional strategies [73], as they would when developing new debriefing approaches. Finally, it is important to keep psychological safety in mind when addressing others’ emotions in a group setting. Importantly, interpersonal emotional regulation is neither psychotherapy nor a mental health intervention. In situations where learners are experiencing significant distress or trauma during a simulated session, the educator’s role is to facilitate access to mental health support rather than attempt to treat the mental health episode ([78].

### Reassess emotional state and determine alignment with learning goals

**Fig. 3:**
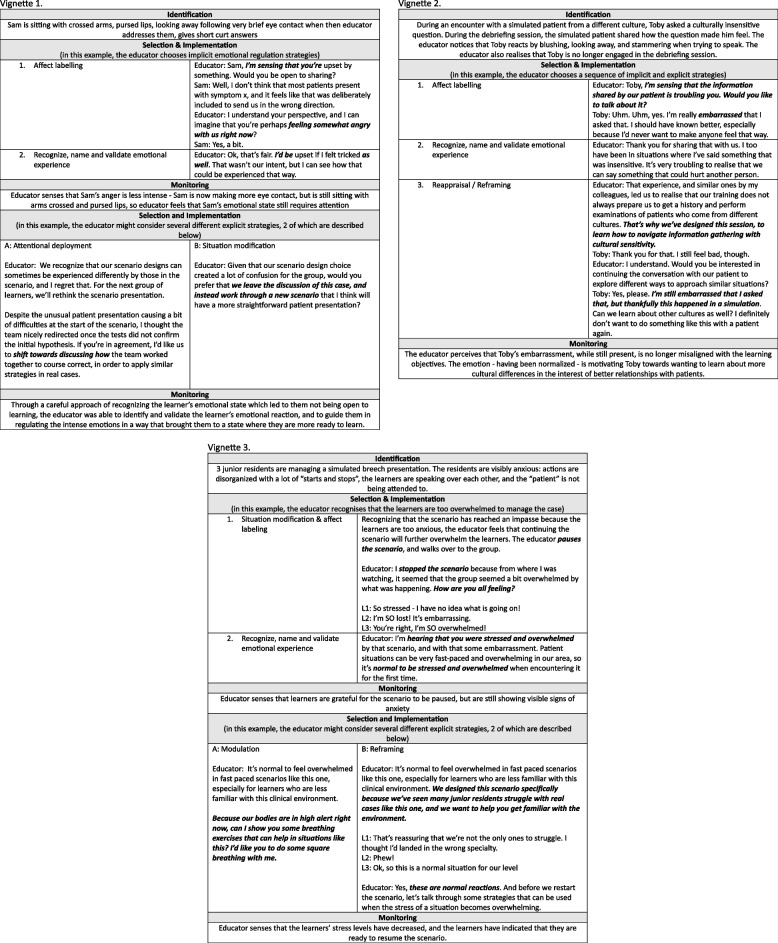
Vignettes demonstrating the application of interpersonal emotional regulation strategies

After implementing a selected strategy, educators should monitor the situation to determine if the strategy is working, and whether to maintain, switch, or stop the attempt at interpersonal emotional regulation. In some circumstances, more than one sequential strategy will be required (e.g., attentional deployment to downregulate strong negative emotions followed by reappraisal) [72, 92]*.* Readers are invited to consult Fig. 3 to see contextualized vignettes in which various strategies could be applied.

## Interpersonal emotional regulation strategies

In this section, we present examples of implicit (more automatic, less effortful) and explicit (more effortful and conscious) interpersonal emotional regulation strategies that simulation educators can use when the learner(s) emotional states are not conducive to the learning goals*.* We strongly emphasize that these strategies should not be employed merely when an emotional state is uncomfortable for the learner and/or the educator. It should have an instrumental purpose—seeking to regulate someone else’s emotions because their current state is not conducive to learning. Situations that potentially warrant interpersonal emotional regulation include—among others—those where a learner’s emotion distracts their attention towards aspects other than what is being discussed (e.g., learner is ruminating on missed intubation and not participating in a discussion about team communications), situations where learners’ emotions overwhelm them and they are not able to attend to the conversation (e.g., learner is angry at the way a symptom was presented during the scenario, and refuses to engage during the debriefing), or situations where learners’ reactions are mismatched to a situation (e.g., learners are rejoicing about their technical prowess, but their interpersonal interactions caused distress for the simulated patient).

### Implicit emotional regulation strategies

A person’s emotional state can elicit matching emotional responses in others, a phenomenon called *emotional contagion *[79]. The emotional expressions of others convey important information about a situation (e.g., “things are good”, or “things are bad”), and can be used as information about how the situation should be interpreted [80]. For example, if one member of the team is showing anxiety, the others could explicitly interpret this as an indication that the situation is more dire than they originally thought [81]. Emotional contagion can also happen unconsciously, where we can “catch”, or be “infected” by another person’s visible emotion through mimicry (also called affective empathy) [82]. Therefore, the emotions expressed by the simulation educator—at any stage of a simulation session—can influence the learners’ own reactions to the simulation session. Practically, this implies educators being mindful of their own emotional state during simulation sessions (e.g., are they arriving at the simulation session angry about something that happened previously; are they anxious because a learner is having an unexpected emotional reaction). Similarly, during emotional situations, whether simulated or real, individuals could use this strategy to “infect” other team members (e.g., a team leader explicitly taking on a calm demeanor with a team that is too emotionally activated, or explicitly manifesting anxiety if the severity of a situation is not fully grasped by the team).

Most people assume that they would feel more distressed if they merely focused on, and talked about, their unpleasant feelings [83]. However, in many situations, focusing on our emotions can dampen them. The action of naming the emotions felt, called “affect labeling”, can serve as a form of implicit emotional regulation [84, 85]. Affect labeling consists of either labeling one’s own emotions (“I feel anxious”) or an aspect of a situation that triggered the emotion (“That scenario was stressful”)[84]. In addition to dampening an emotional experience, affect labeling can decrease the impact of the emotion on immediate as well as subsequent performance[86–88].

In situations where the emotions are high intensity, emotional contagion and affect labeling may diffuse the emotion somewhat, but it is unlikely to completely diffuse it. Also, there are circumstances—such as if the learners are feeling anger or shame—where labeling the emotions will either have no effect or may increase the intensity of the emotions experienced [33, 84, 89–91]. In such situations, facilitators will need to determine whether the participants’ emotional states are conducive to the goals of the situation or not. For example, if a learner is angry at having missed a diagnosis, but is motivated to learn what went wrong and how to avoid it in the future, a facilitator may simply opt to validate the learner’s self-anger and support their desire to learn from the situation. If the emotional states are not conducive to the learning goals, explicit emotional regulation strategies can be used.

### Explicit emotional regulation strategies

The following explicit interpersonal emotional regulation are effortful and conscious strategies that can be used to influence the intensity and course of another person’s emotional state: situation modification, attentional deployment, reappraisal, and modulation.

*Situation modification* consists of changing the situation to which a learner is exposed. This can involve altering a stimulus in terms of its nature, duration, intensity (e.g., making the patient more stable when a learner seems overwhelmed) or introducing or removing stimuli that either change the reaction or trigger the reaction (e.g., adding supports and scaffolding during a scenario to help a learner respond more adaptively to a triggering type of event, letting a learner leave the session for a short break, changing some of the characteristics of a subsequent scenario). Effective strategies can also include distancing from the emotionally provoking situation, but this is not always desirable for learning (e.g., a learner in an acute care specialty needs to be able to function in high-pressure situations) [7, 72, 92].

*Attentional deployment* consists of directing the learner’s attention either towards or away from something [93], and/or selectively attending to one aspect of a situation. This can include distraction (shifting attention from one aspect of a scenario towards another, or entirely away from the situation altogether), concentration (such as focusing on breathing, a sound, or a visual stimulus), and rumination (directing attention inwards towards a feeling and the consequences of the feeling). In general, rumination can be maladaptive, particularly for negative emotions where it can lead to anxiety and depression [71]. Simulation educators can influence what aspects of the situation the learner pays attention to by inviting them to consider a different aspect of the scenario (e.g., more neutral or positive aspect of the scenario). This explicitly directs the learners’ attention to things that are unrelated to what triggered the emotion [92]. This is a strategy that can reinforce self-regulation in learners because it scaffolds and supports the other’s self-regulation rather than fully replacing their efforts [72]. With high-intensity stimuli, attention deployment (other than rumination) can be very effective [94]. For example, if a learner is angry and feeling tricked by a lack of realism, an extended conversation about realism could reinforce the anger and lead the learner to ruminate on the overall lack of realism in simulation. In contrast, encouraging the learner (or group) to consider situations in the real world where key symptoms could be easily missed could successfully redeploy attention to situations less likely to provoke anger.

*Reappraisal* (also called reframing) consists of changing the interpretation of a situation so as to alter its emotional impact [72, 92, 93]. As described below, individuals assign different meanings to a situation by changing how they interpret it, or by exploring another way of managing it [7, 84].

Reappraisal can target the emotion experienced or the situation [72, 92]. When relating to the emotion, this can involve reinterpreting the experience of the emotion itself, such as being reassured that the emotional reaction is normal and healthy. When focused on the situation, reappraisal can involve reinterpreting a negative situation into a positive one (“Isn’t it great that this happened in simulation so that we can learn from it, rather than the first time with a patient?”). Reappraisal can also take the form of perspective-taking, such as using circular questions to encourage reflection on the experience of others in the team [95]. Reappraisal tends to be most effective in lower-intensity situations [96], and if initiated early in the emotional experience [97].

*Modulation* consists of trying to influence how an emotion is expressed, either behaviourally or physiologically [7]. This can involve controlling the outward expression of emotion (e.g., masking one’s emotion) or the internal subjective experience of the emotion (e.g., suppressing any feelings) [93]. When done in an extrinsic manner, examples include asking a learner to calm down or take a deep breath, verbalizing empathy and understanding of what a learner is feeling, as well as physical gestures such as hugs or pats on the shoulders [92]. Threatening contexts and intense negative emotions are more likely to lead people to select response modulation compared to other strategies [72]. Compared to other strategies such as cognitive reappraisal, expressive suppression appears more effective at decreasing a positive emotion but less so for a negative one. In fact, it can sometimes increase the intensity of the negative emotion that one is seeking to diminish [98]. If applied in the wrong way or context, it can also decrease positive relationships [7].

## Conclusions

Simulation-based education can often elicit emotional reactions in participants. These emotions are neither good nor bad; they simply *are*. Their value at any given moment is determined by their utility in meeting the goals of a particular situation. When emotions are particularly intense, or a given emotion is not aligned with the situation, they can impede learners’ ability to engage in a learning session, as well as their ability to retain knowledge and skills learned during the session. In this paper, we have sought to build on existing guidance for educators seeking to optimize the emotional state of learners, by more deeply exploring the theory and research which underpins the practical application. If educators can recognize and identify emotions experienced by others, determine whether those emotional reactions are problematic or helpful for a given situation, and develop the skills to mitigate unhelpful emotions and leverage those that are beneficial in achieving the goals of a simulation session, they are more likely to be able to respond and manage them in ways that are adaptive to the learnings goals of their simulation sessions.

## Data Availability

Not applicable.
